# Non-Hodgkin’s Lymphoma: An Unusual Cause of Shoulder Pain

**DOI:** 10.7759/cureus.36501

**Published:** 2023-03-22

**Authors:** Raymond D.K. Yeak, Yee Yee Yap, Nasir M Nizlan

**Affiliations:** 1 Department of Orthopaedic Surgery, Faculty of Medicine and Health Sciences, Universiti Putra Malaysia, Serdang, MYS; 2 Department of Haematology, Ampang Hospital, Ampang, MYS

**Keywords:** skeletal muscle lymphoma, chemotherapy, extranodal, shoulder pain, non-hodgkin’s lymphoma

## Abstract

Shoulder pain is a common complaint seen in the orthopedic clinic. Here, we report a rare case of primary extranodal ileocecal with exceedingly rare right shoulder deltoid non-Hodgkin’s lymphoma (NHL). A 67-year-old female presented with abdominal swelling for four months associated with loss of appetite, loss of weight, and night sweats. Abdominal contrast-enhanced CT and cecal biopsy confirmed the diagnosis of ileocecal NHL. A right hemicolectomy was performed, and the patient completed six cycles of chemotherapy. The patient developed right shoulder pain with swelling three months later and was diagnosed with a relapse ileocecal lymphoma with dissemination to the right deltoid muscle after a repeat positron emission tomography scan. Clinicians need to consider NHL as a differential diagnosis in evaluating shoulder pain or swelling even though it is exceedingly rare. A partial or non-response to chemotherapy with dissemination to skeletal muscle carries a poor prognosis.

## Introduction

Shoulder pain is a common complaint in elderly patients presenting to the orthopedic clinic. They commonly present with shoulder impingement, rotator cuff tendinopathy or tear, frozen shoulder, or glenohumeral osteoarthritis. Here, we report a rare case of primary extranodal ileocecal with rare right shoulder deltoid non-Hodgkin’s lymphoma (NHL).

## Case presentation

**Figure 1:**
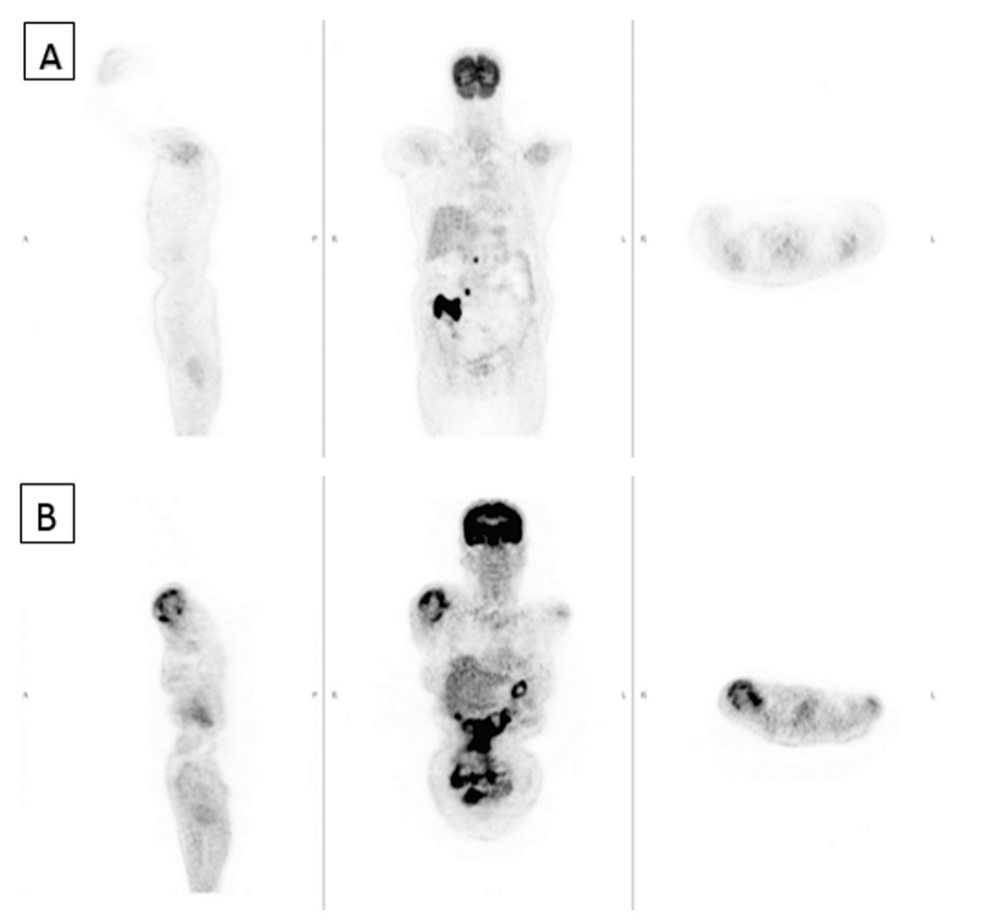
(A) PET scan showing evidence of active lymphoma in the cecum and abdominal and pelvic nodes with no metabolic activity over the right shoulder. (B) Three months later, a PET scan showing FDG hypermetabolism at the right deltoid region associated with FDG-avid active lymphoma in the abdomen and pelvis and a new soft-tissue mass at the right iliac fossa adjacent to the anastomosis site. PET: positron emission tomography; FDG: fluorodeoxyglucose

**Figure 2:**
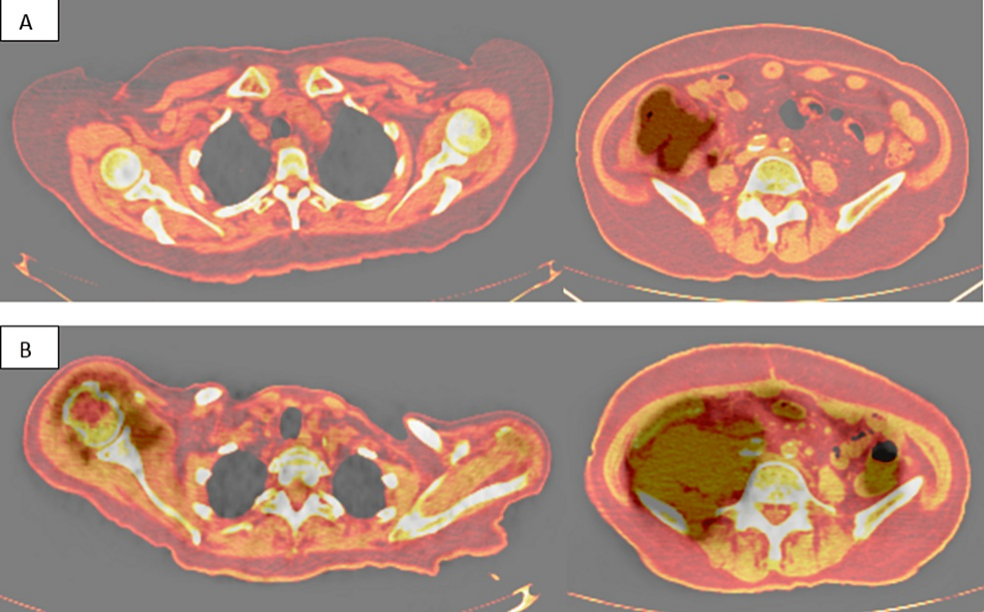
(A) PET scan showing evidence of active lymphoma in the cecum and abdominal and pelvic nodes with no metabolic activity over the right shoulder. (B) Three months later, a PET scan showing FDG hypermetabolism at the right deltoid region associated with FDG-avid active lymphoma in the abdomen and pelvis. PET: positron emission tomography; FDG: fluorodeoxyglucose

A 67-year-old female with a history of type 2 diabetes mellitus on oral metformin 500 mg daily presented with right lower quadrant abdominal swelling for four months associated with loss of appetite, loss of weight of 10 kg, and night sweats. Abdominal contrast-enhanced CT was performed which showed a massive right-sided bowel mass involving the cecum and ascending colon with necrotic mesenteric, para-aortic, and aortocaval lymphadenopathy with local infiltration. Colonoscopy was performed and a cecal biopsy showed a high-grade diffuse large B-cell lymphoma (DLBCL) of the cecum. Bone marrow trephine biopsy showed no evidence of infiltration by the primary disease. A right hemicolectomy was done because of suspected intestinal obstruction and the patient completed six cycles of R-CHOP (rituximab, doxorubicin, cyclophosphamide, vincristine, and prednisone) chemotherapy. A positron emission tomography (PET) scan was performed subsequently (Figures 1A, 2A). Three months later, the patient presented with a fever for five days associated with sudden-onset right shoulder pain with swelling. The ultrasound showed right subacromial-subdeltoid bursitis. A PET scan was done which showed fluorodeoxyglucose (FDG) hypermetabolism at the right deltoid region associated with FDG-avid active lymphoma in the abdomen and pelvis and a new soft tissue mass at the right iliac fossa adjacent to the anastomosis site (Figures 1B, 2B). The patient was started on another cycle of chemotherapy and completed 10 days of chlorambucil and prednisolone. A repeat bone marrow trephine biopsy showed no evidence of infiltration by the primary disease. The patient succumbed to her disease and passed away three months after the onset of the right shoulder swelling.

## Discussion

Lymphoma is a liquid malignant tumor that arises from lymphocytes and is classified as either Hodgkin’s lymphoma or NHL. NHL commonly involves the gastrointestinal tract, Waldeyer’s ring, skin, lung, bone, and the central nervous system. Gastrointestinal NHL accounts for 4-12% of all NHLs [1]. Among the NHL, primary ileocecal NHL is the second most common after gastric lymphoma [2].

Our patient was at the modified Ann Arbor stage IIE2, Paris TNMB system T2, N1, M0, B0, and Lugano stage 2B based on the abdominal contrast-enhanced CT and cecal biopsy. Post-chemotherapy PET scan showed disease progression with evidence of active lymphoma in the cecum, abdomen, and pelvic nodes but the patient chose to watch and wait as she was clinically asymptomatic.

Auger et al. reported a longer survival for patients entering complete remission than those who had a partial or no response with a survival of 34 months versus four months [2]. Three months later, our patient developed a right shoulder swelling which raised the suspicion of skeletal muscle lymphoma due to the underlying ileocecal lymphoma. Tissue biopsy should always be pursued to determine the cause of any abnormal musculoskeletal swelling.

Samuel et al. reported that the presenting history is short, usually of limb swelling developing rapidly over a few weeks [3]. This correlated with our patient who had a sudden onset of shoulder swelling with rapid progression.

Histologically, diffuse large cell NHL is the most common histological subtype for extranodal lymphoma affecting the skeletal muscle [4]. A PET scan was repeated when the patient developed the right shoulder swelling. It showed progression which had disseminated to the right deltoid muscle. The modified Ann Arbor stage at this juncture was stage IVE. The relapse coupled with a high-grade B-cell lymphoma as well as dissemination to the skeletal deltoid muscle worsened her prognosis. Jeffrey et al. reported that lymphoma arising or disseminating to the skeletal muscle had a poor outcome [5]. Primary skeletal muscle involvement by DLBCL is exceedingly rare, comprising less than 0.5% of cases [6]. The Scotland and Newcastle Lymphoma Group has data accumulated over 15 years on over 6,000 patients with NHL and only found eight patients with primary NHL of the muscle [3].

A PET scan is usually performed for staging purposes as well as to monitor response to chemotherapy. Although a PET scan cannot establish the diagnosis, it is a useful adjunctive tool. In our case, the patient had a pre-existing ileocecal lymphoma, therefore, chemotherapy was immediately started for the relapse and skeletal muscle lymphoma after the repeat PET scan. We did not perform a tissue biopsy for the deltoid muscle as the patient had a relapse and the treatment would not differ for both the relapse and the skeletal muscle lymphoma.

## Conclusions

This case report highlights the need for clinicians to consider secondary skeletal muscle NHL as a differential diagnosis while evaluating shoulder pain or swelling even though it is rare. A partial or non-response to chemotherapy with distant metastasis in the elderly will lead to a poorer prognosis.
